# Identification of a Potential PGK1 Inhibitor with the Suppression of Breast Cancer Cells Using Virtual Screening and Molecular Docking

**DOI:** 10.3390/ph17121636

**Published:** 2024-12-05

**Authors:** Xianghui Chen, Zanwen Zuo, Xianbin Li, Qizhang Li, Lei Zhang

**Affiliations:** 1 School of Medicine, Shanghai University, Shanghai 200444, China; 2 Department of Pharmaceutical Botany, School of Pharmacy, Naval Medical University, Shanghai 200433, China; 3 Innovative Drug Research Center, College of Life Sciences, Huaibei Normal University, Huaibei 235000, China; 4 School of Computer and Big Data Science, Jiujiang University, Jiujiang 332000, China; 5 Key Laboratory of Plant Secondary Metabolism and Regulation of Zhejiang Province, College of Life Sciences and Medicine, Zhejiang Sci-Tech University, Hangzhou 310018, China

**Keywords:** breast cancer, prognostic model, phosphoglycerate kinase 1 (PGK1), virtual screening

## Abstract

Background/Objectives: Breast cancer is the second most common malignancy worldwide and poses a significant threat to women’s health. However, the prognostic biomarkers and therapeutic targets of breast cancer are unclear. A prognostic model can help in identifying biomarkers and targets for breast cancer. In this study, a novel prognostic model was developed to optimize treatment, improve clinical prognosis, and screen potential phosphoglycerate kinase 1 (PGK1) inhibitors for breast cancer treatment. Methods: Using data from the Gene Expression Omnibus (GEO) database, differentially expressed genes (DEGs) were identified in normal individuals and breast cancer patients. The biological functions of the DEGs were examined using bioinformatics analysis. A novel prognostic model was then constructed using the DEGs through LASSO and multivariate Cox regression analyses. The relationship between the prognostic model, survival, and immunity was also evaluated. In addition, virtual screening was conducted based on the risk genes to identify novel small molecule inhibitors of PGK1 from Chemdiv and Targetmol libraries. The effects of the potential inhibitors were confirmed through cell experiments. Results: A total of 230 up- and 325 down-regulated DEGs were identified in HER2, LumA, LumB, and TN breast cancer subtypes. A new prognostic model was constructed using ten risk genes. The analysis from The Cancer Genome Atlas (TCGA) indicated that the prognosis was poorer in the high-risk group compared to the low-risk group. The accuracy of the model was confirmed using the ROC curve. Furthermore, functional enrichment analyses indicated that the DEGs between low- and high-risk groups were linked to the immune response. The risk score was also correlated with tumor immune infiltrates. Moreover, four compounds with the highest score and the lowest affinity energy were identified. Notably, D231-0058 showed better inhibitory activity against breast cancer cells. Conclusions: Ten genes (ACSS2, C2CD2, CXCL9, KRT15, MRPL13, NR3C2, PGK1, PIGR, RBP4, and SORBS1) were identified as prognostic signatures for breast cancer. Additionally, results showed that D231-0058 (2-((((4-(2-methyl-1*H*-indol-3-yl)-1,3-thiazol-2-yl)carbamoyl)methyl)sulfanyl)acetic acid) may be a novel candidate for treating breast cancer.

## 1. Introduction

Breast cancer is the most prevalent cancer among women [1]. Breast cancer exhibits high heterogeneity and is classified into four subtypes (luminal A, luminal B, HER-2 enriched (HER2+), and triple-negative (TN) breast cancer) [2]. As a result, the clinical management of breast cancer subtypes, including diagnosis, treatment, and prognosis, is different. Luminal breast cancer (luminal A and B) comprises approximately 70% of breast cancer cases. The HER2+ subtype is sensitive to targeted therapies. TN breast cancer accounts for about 10–15% of breast cancer cases and is the most aggressive subtype. Nonetheless, early detection, diagnosis, and treatment can improve cure rates and reduce mortality rates related to breast cancer. Additionally, understanding breast cancer can improve therapeutic outcomes for patients. Also, accurate and reliable novel molecular biomarkers are essential for predicting the prognosis of breast cancer patients.

The advancement of sequencing technology has allowed for the collection and storage of extensive gene expression data from different molecular subtypes of breast cancer. This information is available in various databases, including the Gene Expression Omnibus (GEO) and The Cancer Genome Atlas (TCGA). The microarray data in the database reflects the general conditions of expression information targeting several genes. Microarray analysis can be used to acquire the expression information and explore differentially expressed genes (DEGs) during the occurrence and development of diseases based on bioinformatics techniques [3]. Furthermore, the role of DEGs in the development of various cancers, such as gastric cancer [4], craniopharyngioma [5], bladder cancer [6], etc., has been studied using bioinformatics techniques. After DEGs screening, some essential genes are identified as potential biomarkers that provide physicians with additional clinical insights. Some key genes can be identified as potential biomarkers through DEGs screening. Several potential biomarkers for triple-negative [7,8], HER2+ [9,10], and luminal [11] breast cancer have been detected via DEGs screening. Moreover, several key DEGs have been identified between different breast cancer subtypes [12,13]. Nevertheless, the characteristic signature genes targeting the four basic breast cancer subtypes have not been detected. Therefore, a prognostic model can be used to determine the overall risk of death for breast cancer patients, thus optimizing treatment and improving clinical prognosis. Although surgery, chemoradiotherapy, and targeted therapy can improve the long-term survival of some breast cancer patients, many patients have poor long-term efficacy and are prone to relapse, mainly due to drug resistance [14]. Therefore, effective prognostic markers and new practical therapeutics are needed based on these markers to enhance breast cancer treatment.

In the present study, a robust risk signature commonly found in four molecular subtypes of breast cancer was developed via integrated bioinformatics analysis. The key DEGs in each subtype were identified via the RRA method. Ten risk genes were screened using Least Absolute Shrinkage and Selection Operator (LASSO) regression analysis and multivariate Cox regression analysis. Based on these genes, a prognostic risk model was then developed. Kaplan–Meier (KM) survival analysis showed that the prognostic model had robust predictive performance. The accuracy of the model was confirmed via receiver operating characteristic (ROC) curve analysis. A nomogram was constructed to facilitate the use of the risk-prognosis model. Notably, phosphoglycerate kinase 1 (PGK1) is believed be an effective therapeutic target [15]. Therefore, novel PGK1 inhibitors were detected via molecular docking and virtual screening.

## 2. Results

### 2.1. DEGs Identification and Functional Enrichment Analysis

**Figure 1:**
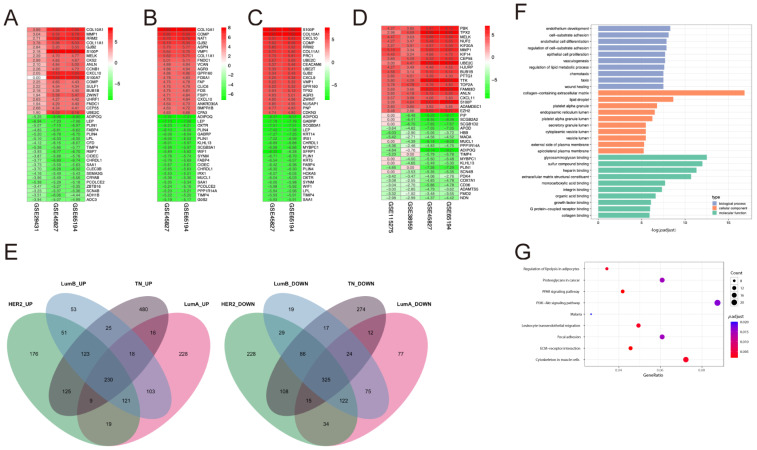
Identification and functional enrichment analysis of DEGs. (**A**–**D**) Top 20 up-regulated and down-regulated genes in HER2 (**A**), LumA (**B**), LumB (**C**), and TN (**D**) subtype tumor samples from GSE29431, GSE38959, GSE45827, GSE65194, and GSE115275 datasets. The red color represents up-regulated genes, while green indicates down-regulated genes. The numbers shown in the figure represent the log fold change (logFC) of genes in each dataset. The cutoff criteria are *p* < 0.05 and |logFC| > 0.5. (**E**) The Venn diagram of DEGs of HER2, LumA, LumB, and TN subtype tumor samples from GSE29431, GSE38959, GSE45827, GSE65194, and GSE115275 datasets. (**F**) The bar plot of GO functional enrichment analysis. The top 10 terms of biological process, cellular component, and molecular function are shown. (**G**) The bar plot illustrates the results of KEGG functional enrichment analysis.

Five gene expression datasets containing normal samples of HER2, LumA, LumB, and TN subtype tumor samples were included after conducting a search in the GEO database. DEGs between the breast tumor samples and the corresponding normal samples were then identified (Table S1). RRA analysis was conducted to identify differentially expressed genes from different datasets (*p* < 0.05 and |logFC| > 0.5). A total of 1801, 1428, 1421, and 1887 DEGs were found in HER2, LumA, LumB, and TN groups, respectively. The top 20 up-regulated and down-regulated genes consistently expressed in the different profiles are shown in Figure 1A–D. The Venn plots demonstrated that 230 up- and 325 down-regulated DEGs were common across the four subtypes (Figure 1E). Functional enrichment analysis using gene ontology (GO) terms and pathway enrichment analysis based on the Kyoto Encyclopedia of Genes and Genomes pathway (KEGG) was conducted for these genes. GO enrichment analysis identified 511 terms related to the DEGs, including 453 biological process (BP) terms, 26 cellular component (CC) terms, and 31 molecular function (MF) terms (Tabel S3). The top 10 BP, CC, and MF terms are shown in Figure 1F (endothelium development; cell–substrate adhesion; and endothelial cell differentiation in BP; collagen-containing extracellular matrix; lipid droplet; and platelet alpha granule in CC; and glycosaminoglycan binding; sulfur compound binding; and heparin-binding in MF). KEGG pathway analysis revealed nine pathways related to the DEGs (Tabel S4), including extracellular matrix (ECM)-receptor interaction, peroxisome proliferator-activated receptor (PPAR), leukocyte transendothelial migration, regulation of lipolysis in adipocytes, cytoskeleton organization in muscle cells, focal adhesion, proteoglycans in cancer, the PI3K-Akt signaling pathway, and malaria signaling pathways (Figure 1G).

### 2.2. Independent Prognostic Model Analysis

**Figure 2:**
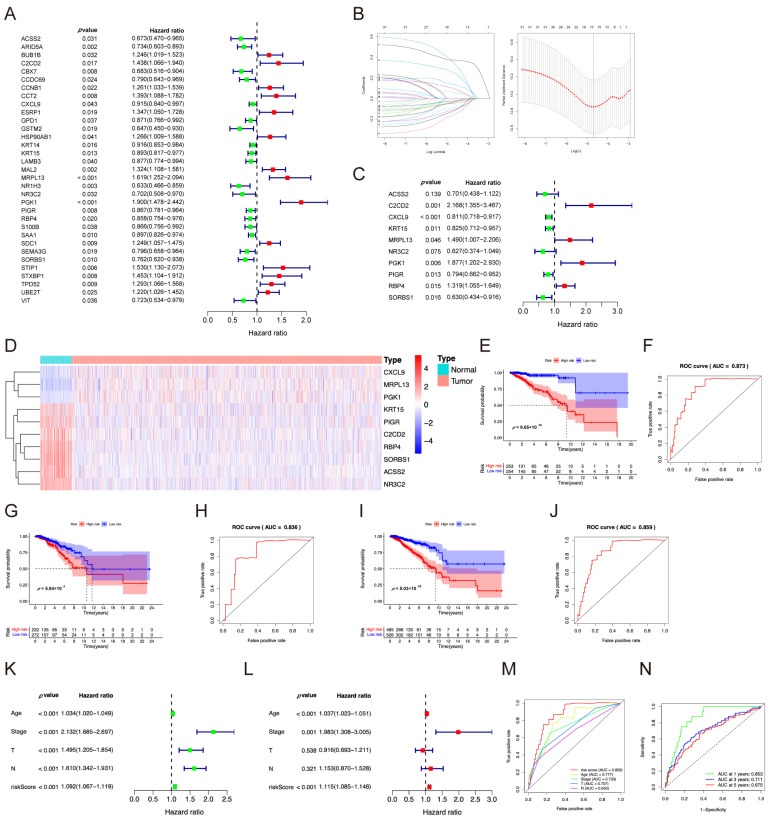
Analysis of the prognostic model in BC. (**A**) Forest plot of the signature risk model. (**B**) Lasso model for screening the key genes. (**C**) Multivariate Cox analysis confirming hub genes for risk model. (**D**) The expression levels of ten hub genes in breast cancer tissues compared to normal tissues. (**E**,**G**,**I**) Kaplan–Meier analysis of survival differences between high-risk and low-risk groups in training (**E**), test (**G**), and entire (**I**) sets. (**F**,**H**,**J**) Receiver operating characteristic (ROC) curve analysis on the ten model gene signatures in the training (**F**), test (**H**), and entire (**J**) sets. AUC, the area under the curve. These curves are performed by R package survival ROC. (**K**) Univariate Cox analysis of risk score and clinicopathological features in the entire set. (**L**) Multivariate Cox analysis of clinicopathological features and risk score in the entire set. (**M**) The ROC curve of the risk score and clinical characteristics. (**N**) The ROC curve and AUC values for the predictive signature at 1-year, 3-year, and 5-year survival rates.

The RNA-seq dataset and clinical information from TCGA were combined to obtain more information. Patients who survived for less than 30 days and lacked the required data were excluded. The patients were divided into a training set (to identify possible prognostic genes) and a test set (for validation). Univariate Cox regression analysis revealed that 33 DEGs, comprising 14 high- and 19 low-risk genes, were significantly associated with survival (*p* < 0.05) (Figure 2A). LASSO regression analysis was performed, and a 15-gene signature was created (Figure 2B). Multivariate Cox regression identified ten genes, including four high- and six low-risk genes (ACSS2 (acyl-CoA synthetase short-chain family member 2), C2CD2 (C2 calcium-dependent domain containing 2), CXCL9 (C-X-C motif chemokine ligand 9), KRT15 (keratin 15), MRPL13 (mitochondrial ribosomal protein L13), NR3C2 (nuclear receptor subfamily 3 group c member 2), PGK1 (phosphoglycerate kinase 1), PIGR (polymeric immunoglobulin receptor), RBP4 (retinol-binding protein 4), and SORBS1 (sorbin and SH3 domain-containing 1)) (Table 1, Figure 2C). The heatmap of gene expressions from TCGA is shown in Figure 2D. The gene levels from GEO datasets were expressed as boxplots in Figure S1. Risk scores were calculated using expression levels and coefficients of 10 genes: Risk score = (−0.3552 × ACSS2 expression) + (0.7737 × C2CD2 expression) + (−0.2091 × CXCL9 expression) + (−0.1919 × KRT15 expression) + (0.3990 × MRPL13 expression) + (−0.4672 × NR3C2 expression) + (0.6295 × PGK1 expression) + (−0.2308 × PIGR expression) + (0.2766 × RBP4 expression) + (−0.4613 × SORBS1 expression). The patients in the training set were categorized into high- and low-risk groups according to the median prognostic risk score (0.9669) to assess the prognostic value of the risk signature. The KM survival curve demonstrated that the survival rate was lower in the high-risk group compared to the low-risk group (*p* = 9.65 × 10^−10^; Figure 2E), indicating that the risk score effectively predicted the prognosis of breast cancer patients.

The prognostic ability of the model was evaluated using the area under the curve (AUC) of the ROC. The model had high sensitivity and specificity (AUC value, 0.873) in the training set (Figure 2F). The model was validated using the test and entire datasets, and similar results were obtained (Figure 2G–J and Figure S2). Univariate Cox regression analysis related to clinical characteristics revealed that age, tumor status, T status, N status, and risk score were significantly associated with overall survival (OS) in the complete dataset (Figure 2K). Furthermore, multivariate Cox analysis showed that age, tumor status, and risk score had excellent independent prognostic value (Figure 2L).

The accuracy of the model and predicting the 1-, 3-, and 5-year overall survival was assessed via the ROC curve. The AUC of the risk score model was 0.859, outperforming clinical characteristics (Figure 2M), demonstrating the robust predictive capability of the model. Furthermore, the AUC values of the model in predicting 1-, 3-, and 5-year survival were 0.853, 0.711, and 0.675, respectively, indicating a good predictive ability of the prognostic model (Figure 2N). The model performed better when the area under the curve was larger. The findings indicate that the developed prognostic risk model can be used to assess breast cancer prognosis.

### 2.3. Correlation Between Risk Score and Clinical Characteristics of Breast Cancer Patients

**Figure 3:**
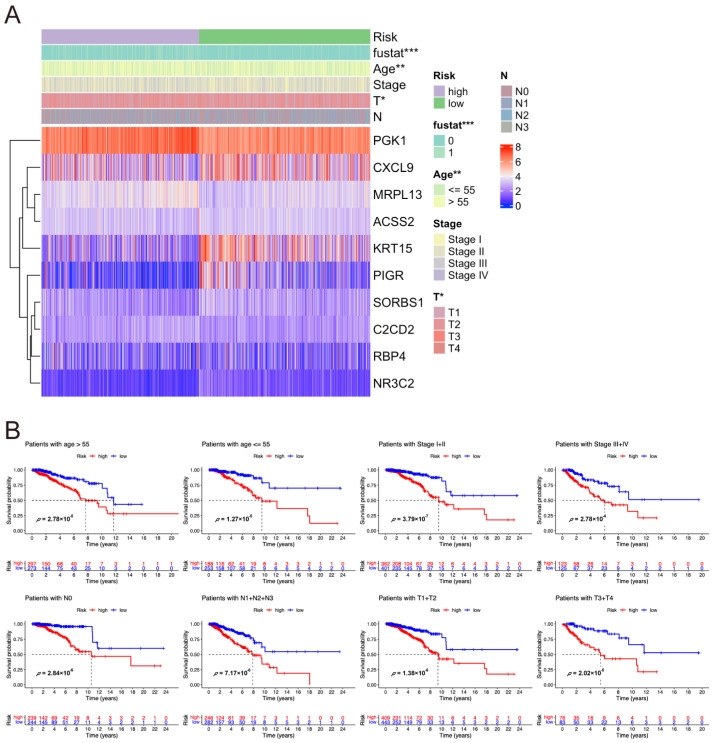
Analysis of the relationships between risk score and clinical characteristics of breast cancer in the TCGA cohort. (**A**) Heat map of ten model genes and clinical characteristics in the high- and low-risk groups. *, *p* < 0.05; **, *p* < 0.01; and ***, *p* < 0.001. (**B**) Analysis of overall survival in TCGA-BC patients based on clinical stratification, focusing on high- and low-risk groups by age, clinical stage, N stage, and T stage.

The clinicopathological characteristics, including survival state, age, and T stage, were compared between the two risk groups. Survival state (*p* < 0.001), age (*p* < 0.01), and T stage (*p* < 0.05) were significantly different between the high- and low-risk groups (Figure 3A). Meanwhile, gene expression patterns were determined to assess the correlation between the genes in the model and risk scores. The results showed that PGK1, MRPL13, and C2CD2 were overexpressed in the high-risk group, while ACSS2, CXCL9, KRT15, NR3C2, PIGR, RBP4, and SORBS1 were highly expressed in the low-risk group. Furthermore, survival analysis of the entire TCGA cohort demonstrated that the low-risk group had a significantly better prognosis than the high-risk group across all clinically stratified subgroups, including age, clinical stage, N stage, and T stage (Figure 3B).

### 2.4. Nomogram Construction

**Figure 4:**
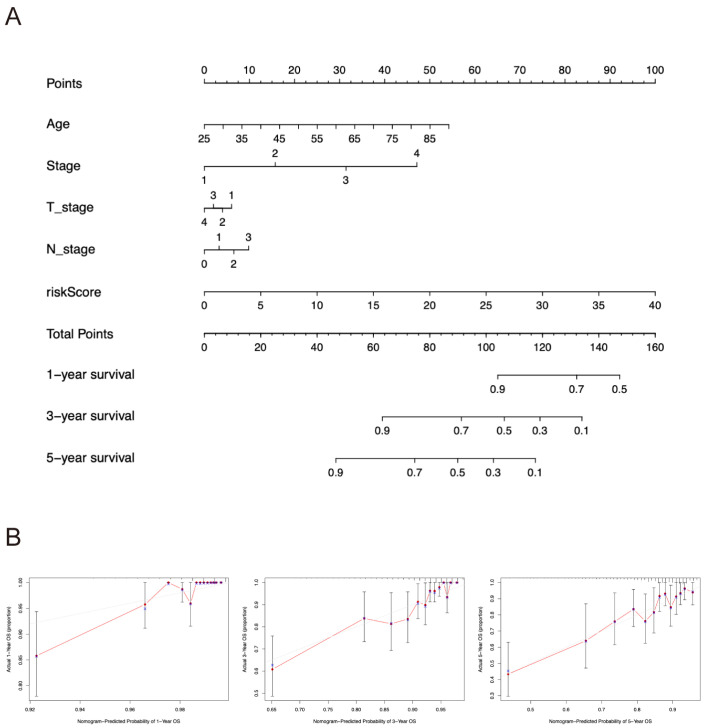
The nomogram in predicting overall survival of breast cancer. (**A**) The nomogram predicts 1-, 3-, and 5-year overall survival. (**B**) Calibration maps were utilized to predict survival rates at 1, 3, and 5 years.

Nomogram is widely used to quantify the results of Cox regression equations [16]. Herein, a nomogram was developed to predict the 3-year, 5-year, and 10-year overall survival status of breast cancer patients based on independent prognostic analysis (Figure 4A). Age, clinical stage, T and N stages, and risk score were treated as variables. The total score was calculated by adding the score of each factor which has a specific score (according to the regression coefficient). A higher score value indicates a higher mortality risk and poorer patient survival. The prediction model was calibrated via calibration curves (Figure 4B). The calibration lines were close to the optimal calibration lines, indicating that the model had good predictive accuracy in specific years.

### 2.5. Functional Enrichment Analyses

**Figure 5:**
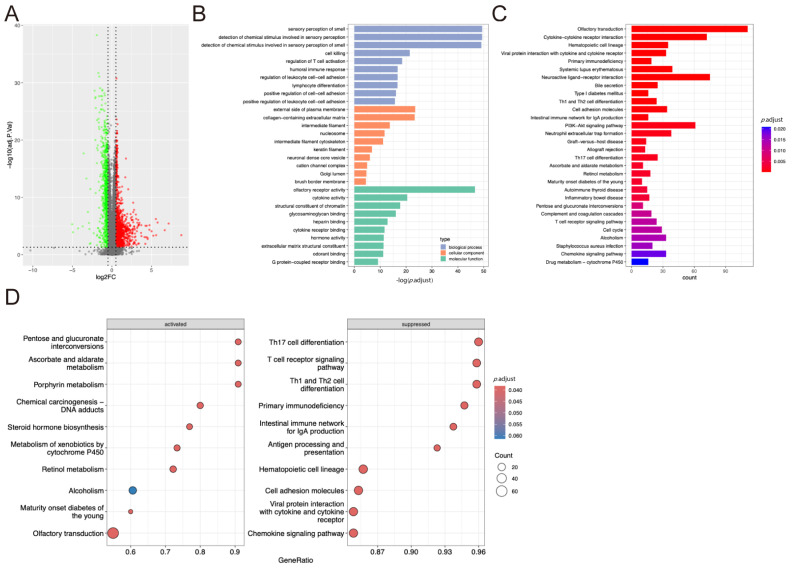
Analysis of functional enrichment across different risk groups. (**A**) Volcano chart of differentially expressed genes; (**B**) GO analysis explored the potential function in terms of biological process (BP), cellular component (CC), and molecular function (MF); (**C**) KEGG analysis showed the potential pathway enrichment; (**D**) GSEA analysis demonstrated the potential activated and suppressed pathway enrichment in the high-risk group compared with the low-risk group.

GO and KEGG analyses were conducted to further confirm the biological mechanisms underlying the different prognoses observed between the low- and high-risk groups. A total of 1953 DEGs were found with adjusted *p* < 0.05 and |logFC| > 0.5 between low- and high-risk groups (Figure 5A). GO functional enrichment showed that these DEGs were related to the regulation of T cell activation, humoral immune response, and lymphocyte differentiation for biological process (BP); external side of the plasma membrane, collagen-containing extracellular matrix, and intermediate filament for cellular component (CC); and cytokine activity, structural constituent of chromatin, and cytokine receptor binding for molecular function (MF) (Figure 5B). Furthermore, KEGG showed that the DEGs were related to pathways involving hematopoietic cell lineage, primary immunodeficiency, systemic lupus erythematosus, Th1 and Th2 cell differentiation, and intestinal immune network for IgA production (Figure 5C). Additionally, the GSEA results suggested that these DEGs were mainly related to chemical carcinogenesis, including DNA adducts (normalized enrichment score (NES) = 0.63, adjusted *p* < 0.01), cell cycle (NES = 0.44, *p* < 0.05), T cell receptor signaling pathway (NES = −0.56, *p* < 0.01), and Th1 and Th2 cell differentiation (NES = −0.54, *p* < 0.01) (Figure 5D). These results suggest that these DEGs are associated with the immune response.

### 2.6. Tumor Immune Infiltration Correlation Analyses

**Figure 6:**
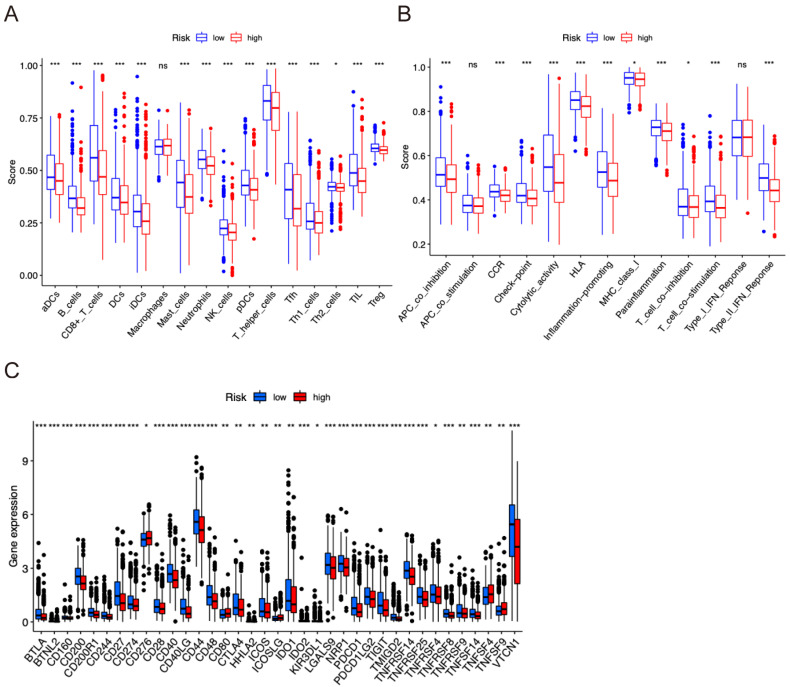
Immune features analysis in risk groups. (**A**,**B**) ssGSEA (single-sample gene set enrichment analysis) scores for immune cells (**A**) and immune function (**B**) in TCGA cohort. (**C**) The expression of immune checkpoint-related genes and the correlation between risk scores. aDCs, activated dendritic cells; APC, antigen-presenting cell; CCR, chemokine receptor; HLA, human leukocyte antigen; iDCs, immature dendritic cells; IFN, interferon; MHC, major histocompatibility complex; NK, natural killer; pDCs, plasmacytoid dendritic cells; Tfh, T follicular helper; Th, T helper cell; TIL, tumor-infiltrating lymphocyte; Treg, T regulatory cell. * *p* < 0.05; ** *p* < 0.01; *** *p* < 0.001; ns, non-significant.

The relationship between prognostic risk and the infiltration of immune cells was also assessed. Compared with the low-risk group, the infiltration scores of most immune cells activated dendritic cells (aDCs), B cells, CD8+ T cells, dendritic cells (DCs), interdigitating dendritic cells (iDCs), mast cells, neutrophils, natural killer (NK) cells, plasmacytoid dendritic cells (pDCs), T helper cells, T follicular helper (Tfh), T helper (Th) 1 cells, Th2 cells, tumor-infiltrating lymphocytes (TIL), and regulatory T cells (Treg) were significantly decreased in the high-risk group (*p* < 0.05; Figure 6A). In addition, levels of APC_co-stimulation, CCR, check-points, cytolytic activity, HLA, inflammation-promoting factors, MHC_class_I, parainflammation, T_cell_co-inhibition, T_cell_co-stimulation, and Type_II_IFN_Response were higher in the low-risk group than in the high-risk group (*p* < 0.05; Figure 6B). Further results showed that ICB expression increased in the low-risk subgroup compared to the high-risk group (Figure 6C). These findings indicate that immune activation and immune status are significantly different between the two groups. Specifically, the low-risk group exhibited a stronger immune microenvironment and a better prognosis than the high-risk group. Therefore, patients in the high-risk group may have weakened immunity, which may result in a lower responsiveness to immunotherapy.

### 2.7. Identification of Novel Inhibitors of PGK1

**Figure 7:**
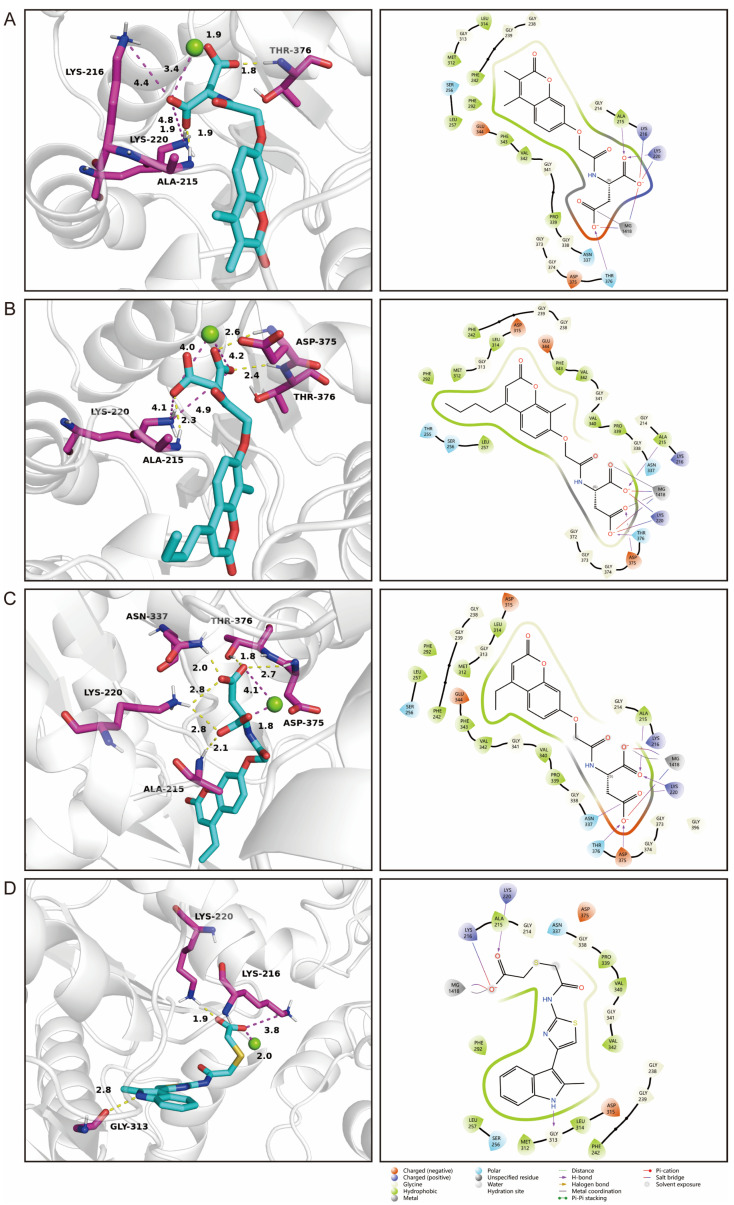
Three-dimensional interaction between PGK1 (2X13) and D715-2871 (**A**), Y040-8304 (**B**), D715-0344 (**C**), and D231-0058 (**D**). Yellow dotted lines represent hydrogen bonds, pinkish-red dotted lines represent salt bridges, and green balls depict magnesium ions.

The therapeutic potential for targeting the risk genes was investigated. Studies have shown that PGK1 is a promising therapeutic target for breast cancer treatment [17]. Herein, compounds that can bind to PGK1 were detected through virtual screening. A total of 1,620,219 compounds were screened from Chemdiv and Targetmol libraries via High Throughput Virtual Screening (HTVS) mode of Glide. The top 10% of compounds were then screened phase in SP mode. Next, 1267 compounds were identified in the XP mode. The rescoring of the relative binding affinity of ligand and protein was performed by MMGBSA (molecular mechanics generalized born surface area). The four best compounds with the highest score and the lowest affinity energy (<−16 kcal/mol) were selected based on the Quantitative Estimate of Drug-likeness (QED) value, pan-assay interference compounds, and docking scores (Table 2). D715-2871 formed two hydrogen bonds (bond lengths: 1.8 Å and 1.9 Å) with three amino acids (Thr376 and Ala215), four salt bridges, and a coordinate bond (Figure 7A). Y040-8304 formed three hydrogen bonds with three amino acids (Ala215, Thr376, and Asp375), four salt bridges, and two coordinate bonds (Figure 7B). D715-0344 formed six hydrogen bonds with five amino acids (Ala215, Lys220, Asp375, Thr376, and Asn337), two salt bridges, and a coordinate bond (Figure 7C). D231-0058 formed five non-covalent bonds with PGK1, including two hydrogen bonds, two salt bridges, and one coordinate bond (Figure 7D).

### 2.8. D231-0058 Inhibits the Growth of Breast Cancer Cells

**Figure 8:**
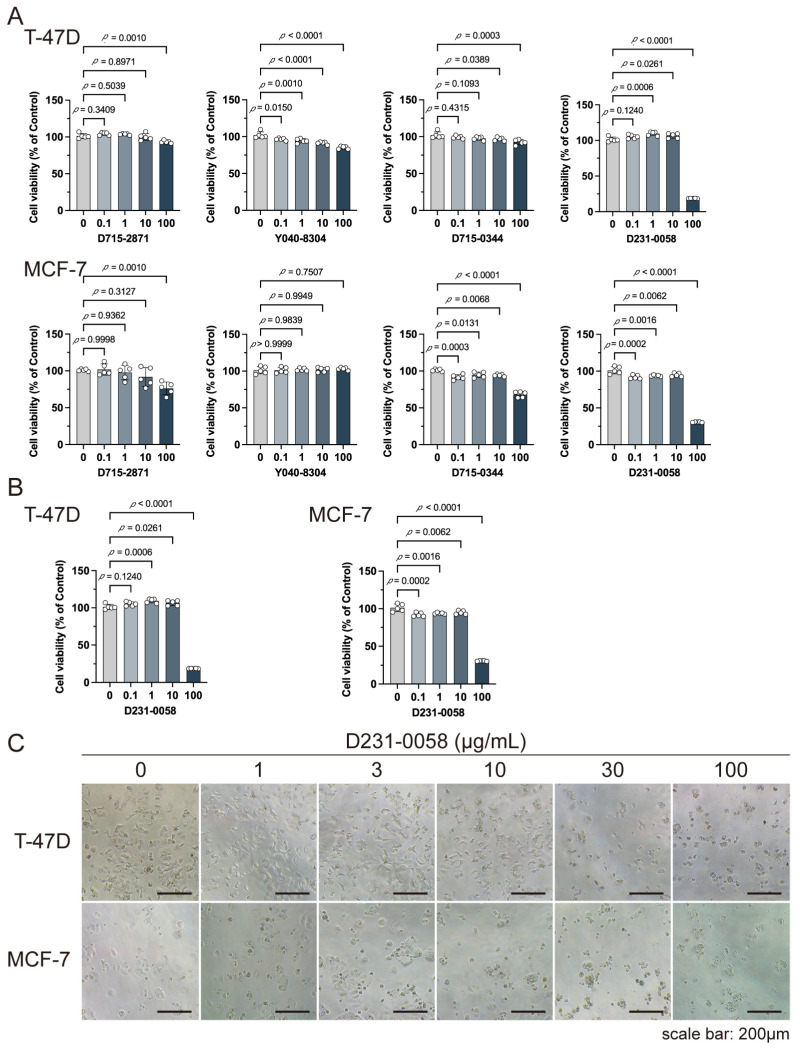
Inhibitory activity of D715-2871, Y040-8304, D715-0344, and D231-0058 against breast cancer cells T-47D and MCF-7. (**A**) CCK8 assay for cell viability. Cancer cells were treated with D715-2871, Y040-8304, D715-0344, or D231-0058 (0, 0.1, 1, 10, and 100 μg/mL) for 24 h. Data were presented as mean ± SD (*n* = 6). (**B**) CCK8 assay for cell viability. Cancer cells were treated with D231-0058 (0, 1, 3, 10, 30, and 100 μg/mL) for 24 and 48 h. Data were presented as mean ± SD (*n* = 6). (**C**) Microscopic observation of the cells treated with D231-0058 (0, 1, 3, 10, 30, and 100 μg/mL) for 24 h.

These compounds were used to treat breast cancer T-47D and MCF-7 cells for validating the antitumor effects of the selected compounds. D715-2871 and Y040-8304 showed weaker inhibition activities, 100 μg/mL D715-0344 showed a better inhibition activity for MCF-7 cells, and D231-0058 exhibited significant inhibitory activity against tumor cells T-47D and MCF-7 (Figure 8A). The half maximal inhibitory concentration (IC_50_) of D231-0058 was determined via CCK8 assays. The IC_50_ values of T-47D cells at 24 and 48 h were similar. The IC_50_ values in MCF-7 cells were 28.66 μg/mL for 24 h and 18.88 μg/mL for 48 h, respectively (Figure 8B). Cell morphology changes were also observed (Figure 8C).

## 3. Discussion

Breast cancer is the most common malignancy among women worldwide, with a high mortality rate. Surgery, chemotherapy, radiation therapy, endocrine therapy, targeted therapy, and immunotherapy are the major treatment methods for breast cancer. Early detection can reduce breast cancer-related mortality [1]. Bioinformatics analyses are widely used for basic tumor research, clinical treatment, and prognostic prediction. Developing polygenic models that target genes for prognosis is promising for the diagnosis, treatment, and preventing cancers. Many prognostic models have been developed for various cancer types, including breast cancer [18,19]. Breast cancer is a highly heterogeneous neoplasm, and thus has different diagnosis, treatment, and prognosis methods. Many models have been constructed for breast cancer subtypes such as HER2+ [20], triple-negative [21], and ER+/HER2− [22]. In the present study, a prognostic model was developed using DEGs.

A single gene expression dataset has limited sample information and thus data from multiple datasets are necessary. Robust rank aggregation (RRA) analysis is widely used to integrate various gene expression datasets and identify critical genes [23]. RRA is used to compare different rankings of genes in an unbiased manner. Zhong et al. identified 194 high-ranking DEGs in triple-negative breast cancer (TNBC) compared with non-TNBC using four GEO datasets of TNBC patients [7]. Sun et al. identified 494 key DEGs in four gene expression profiles from GEO via the RRA method [24]. Herein, DEGs were identified from different subtypes through integrated analysis using the RRA method and Venn analysis for enrichment analysis. KEGG signaling pathway analysis showed that these genes were significantly enriched in ECM–receptor interaction, PPAR signaling pathway, and leukocyte transendothelial migration. The ECM–receptor interaction pathway is found to be a critical signaling pathway involved in promoting breast cancer cell metastasis [25]. The PPAR pathway and leukocyte transendothelial migration are activated in breast cancer [26,27].

LASSO and COX regressions are widely used to establish prognostic models [28,29]. Herein, a ten-gene risk signature, ACSS2, C2CD2, CXCL9, KRT15, MRPL13, NR3C2, PGK1, PIGR, RBP4, and SORBS1, was developed. ACSS2 catalyzes acetyl-CoA synthesis and provides a nucleocytosolic source for acetyl-CoA, thereby playing a key role in epigenetic regulation and metabolic homeostasis [30]. ACSS2 also plays a crucial role in cell growth in various cancers, such as breast cancer [31]. Therefore, ACSS2 is a promising target for inhibitor development [32]. C2CD2 is a prognostic biomarker for breast cancer in bioinformatics [33]. CXCL9, a chemokine, promotes lymphocytic infiltration in solid tumors [34]. CXCL9 overexpression results in T cell accumulation and improved survival in ovarian cancer, acting as a predictive biomarker [35]. Additionally, CXCL9 is a potential clinical biomarker in ER-negative [36] and triple-negative [37] breast cancer. KRT15 is a type I cytoskeletal protein [38]. KRT15 has been detected in the bulges of hair follicles. KRT15 is involved in the growth and differentiation of hair follicles as well as the growth of the lacrimal gland [39,40]. KRT15 has different functions in cancers. KRT15 can promote the migration and invasion of colorectal cancer cells [41]. However, a low expression level of KRT15 is significantly linked to a poorer prognosis in breast cancer [42]. MRPL13, belonging to the MRP family, is involved in mitochondrial protein translation and is essential for mitochondrial bioenergetic and metabolic processes [43]. Many reports have shown that MRPL13 is involved in tumorigenesis [44,45]. MRPL13 promotes cell proliferation, migration, and epithelial–mesenchymal transition (EMT) in breast cancer by activating the PI3K/AKT/mTOR signaling pathway [46]. Also, MRPL13 is a prognostic biomarker [47,48]. NR3C2 is involved in regulating electrolyte balance and blood pressure [49]. In addition, NR3C2 can suppress cell development [50] and is a prognostic target in breast cancer [51,52]. PGK1 is a crucial component involved in the glycolysis process [53]. PGK1 catalyzes 1,3-bisphosphoglycerate to ADP, producing 3-phosphoglycerate and ATP. In addition, studies have demonstrated that PGK1 participates in tumor development. Elevated succinate concentration enhances PGK1 succinylation, inhibiting the degradation of PGK1 and increasing lactate generation [54]. Reduced succinate and lactate levels can also inhibit tumor growth of glioblastoma. PGK1 down-regulation inhibits cell proliferation, invasion, and metastasis in breast cancer in vitro and in vivo [55]. PGK1 is an effective therapeutic target in the diagnosis, prognosis, and treatment of cancers, including breast cancer [15]. PIGR is widely expressed in epithelial cells and functions as a transporter for the transcytosis of polymeric immunoglobulins [56]. Furthermore, PIGR is a biomarker in many types of cancer [57]. Several studies have indicated that PIGR has prognostic value in breast cancer [58,59]. Nevertheless, the functions of PIGR in breast cancer should be further investigated. RBP4 is involved in transporting retinol (vitamin A) and other retinoid derivatives in circulation and is mainly secreted by the liver and adipose tissue [60]. RBP4 is also identified as a biomarker for diagnosing and prognosing hepatocellular carcinoma [61], gastric cancer [62], and breast cancer [63]. The SORBS1 gene, encoding Cbl-associated protein (CAP), plays an important role in insulin signaling [64] and regulates cell proliferation and invasion [65]. SORBS1 reduces cell proliferation, invasion, and migration in breast cancer through the regulation of miR-142-5p [66]. SORBS1 also blocks epithelial–mesenchymal transition by regulating PI3K/AKT signaling [67]. In addition, SORBS1 is an independent prognostic marker for breast cancer [68].

DEGs in breast cancers are majorly enriched in immune-related terms, suggesting that many immune-related signaling pathways are abnormally regulated in breast cancers [69]. Tumor immunotherapy has developed rapidly in recent years. Breast cancer is a non-immunological disease, and immunotherapy has an unsatisfactory effect in the early stages [70,71], which is a big challenge. Therefore, combinatorial strategies are useful in such cases. For example, progranulin (PGRN) is involved in many physiological processes [72] and is also related to poor prognosis [73]. Fang et al. found that PGRN can promote M2 polarization and PD-L1 expression of macrophages by activating the STAT3 pathway, thereby promoting the immune escape of breast tumors through PD-1/PD-L1 interaction [74]. This suggests that the combination of PGRN targeting and PD-L1/PD-1 inhibitors can improve the efficacy of clinical immunotherapy for breast cancer. Therefore, potential targets should be identified to improve breast cancer treatment.

Molecular docking technology is crucial in computer-aided drug design. This technology allows the screening of specific molecules with good drug-forming properties from bulk compound libraries. PGK1 is up-regulated in breast cancer and is a promising therapeutic target [75]. However, only a few PGK1 inhibitors have been reported. PGK1 generated ATP in the glycolytic pathway by converting 1,3-diphosphoglycerate (1,3-BPG) and ADP into 3-phosphoglycerate (3-PG) [76]. Terazosin can occupy the ADP-binding pocket and inhibit PGK1, which is a competitive inhibitor [77]. In one study, two novel small molecules (CHR-6494 and Z57346765) targeting the ADP-binding pocket were identified via virtual screening [78]. Liao et al. identified an ATP-competitive inhibitor of PGK1, DC-PGKI (ethyl 6,7-dichloro-3-(4-((4-(piperazin-1-yl)phenyl)carbamoyl) piperazin-1-yl) quinoxaline-2-carboxylate), through a high-throughput screening platform (IC_50_ value; 0.16 μM) in lung cancer cells [79]. This strategy has also been used in this study (Figure S3). The ADP-binding pocket is crucial for the activity of the metabolic enzyme PGK1. Herein, small-molecule compounds were screened from Chemdiv and Targetmol libraries via virtual screening, and finally, the top four compounds with the highest score and the lowest affinity energy were selected. Interestingly, the top three (D715-2871, Y040-8304, and D715-0344) have the same backbone structure (belonging to a class of compounds distinct from the side chains). The compounds have a 7-hydroxy-coumarin structure. D715-2871 has two methyl groups at C3 and C4 of coumarin, Y040-8304 has a butyl group at C4 and a methyl group at C8, and D715-0344 has an ethyl group at C4. The compounds also have identical 7-OH substituents, a propionamide bonded to an N atom with succinic acid. The carboxyl groups of succinic acid are reactive groups. D231-0058 has a completely different structure from the other three, except for the reactive group. In addition, some non-ATP-competitive inhibitors of PGK1 have been developed. GQQ-792, a thiodiketopiperazine derivative, binds to cysteine residues near the ATP-binding pocket [80]. Ilicicolin H is also a non-ATP-competitive inhibitor and binds to non-ATP binding sites [81]. The binding energy is used to evaluate the docking results where the smaller binding energy indicates a more stable conformation [82]. Z57346765 is a commercial PGK1 inhibitor with a binding energy below −9.515 kcal/mol. In this study, the compounds had lower binding energies, with only D231-0058 exhibiting better inhibitory effects against breast cancer cells. Therefore, the PGK1 inhibitor is a promising candidate for the treatment of breast cancer.

## 4. Materials and Methods

### 4.1. Compounds and Cells

Breast cancer cell lines T-47D and MCF-7 were obtained from the China Center for Type Culture Collection in Wuhan, China. They were cultivated in RPMI1640, supplemented with antibiotics (100 U/mL penicillin and 100 mg/mL streptomycin) and 10% fetal bovine serum. The cells were incubated at 37 °C in a 5% CO_2_ incubator. Compounds Y042-8649, D715-0537, Y043-8503, and D715-0389 were obtained from Tsbiochem (Shanghai, China).

### 4.2. Data Source and Preprocessing

Five microarray datasets (Table S2) were downloaded from the National Center for Biotechnology Information (NCBI) GEO database (https://www.ncbi.nlm.nih.gov/geo/, accessed on 24 March 2016). A total of 357 breast tumor samples and 51 normal breast samples were included in this analysis. Based on the breast cancer subtypes, these samples included 117 HER2, 58 LumA, 60 LumB, and 132 TN tumor samples. In addition, other gene expression profile datasets, GSE32641, GSE36295, GSE42568, GSE53752, and GSE139038, were downloaded to validate the mRNA expression level. The RNA-seq data and corresponding clinical information were obtained from TCGA (https://www.cancer.gov/about-nci/organization/ccg/research/structural-genomics/tcga, accessed on 1 April 2022). A total of 1097 clinical information records were obtained, while 1109 breast cancer samples and 113 normal samples were collected to construct the model.

### 4.3. Differential Expression Analysis and Data Preprocessing

The matrix files were retrieved and analyzed using R (version 3.6.3). The R package limma (version 3.42.0) was adopted to identify DEGs between breast tumor and normal breast samples. The R package RobustRankAggreg (version 1.1) was used for the integrated analysis. The |logFC| > 0.5 and *p* < 0.05 were statistically significant.

### 4.4. Functional Enrichment Analysis

GO functional enrichment and KEGG enrichment analyses were performed using the R packages GO.db (version 3.10.0) and org.Hs.eg.db (version 3.10.0).

### 4.5. Construction of the Prognostic Model

Based on the BC tumor samples and clinical information in the TCGA dataset, the DEGs were subjected to examine the correlation between mRNA expression levels and OS with *p* < 0.05 by univariate Cox regression analysis using R packages survival (version 3.1-12). The LASSO algorithm was used to screen candidate genes. Breast tumor samples were randomly divided into the training set and the test set in a 1:1 ratio. The training set was utilized to build a prognostic model, while the test set and the entire set were employed to validate this model. Multivariate Cox regression analysis was used to screen independent prognostic DEGs with *p* < 0.05 as the threshold of significant correlation. Based on the expression levels of these genes and coefficients (coef) in the multivariate Cox proportional hazards regression analysis, a prognostic risk score formula was defined: Risk score = $\sum_{i=0}^{n}gene\;expression\;level\times coef$. All patients were categorized into low- and high-risk groups based on the median risk score. R package survival (version 3.1-12) was used to construct the KM curve, which was utilized to evaluate the prognostic value of the risk score. The log-rank *p*-value and the hazard ratio (HR), along with their 95% confidence intervals, were also presented. ROC curve analysis generated from R package survivalROC (version 1.0.3) was used to compare the sensitivity and specificity. The AUC was calculated by the ROC curve. Traditional clinical risk factors such as age, gender, tumor status, T status, N status, and risk score were presented by univariate and multivariate Cox analysis. The nomogram was created using multivariate Cox analysis with the R package rms (version 6.1-1).

### 4.6. Molecular Docking

The crystal structure of human PGK1 was obtained from the RCSB Protein Data Bank (PDB, 2X13) and has a resolution of 1.74 Å. The hydrogen atoms and charges were added into the protein structure using the Protein Preparation Wizard in the Schrödinger suite, which was then used for computational screening techniques. Chemdiv and Targetmol libraries (including more than 1620 thousand compounds) were selected to identify potential PGK1 inhibitors. For the virtual screening process, the High Throughput Virtual Screening (HTVS) protocol within the Glide module was initially employed, followed by Standard Precision (SP) docking of the top 10% of ligands from the HTVS based on their docking scores. Finally, Extra Precision (XP) docking was performed on the top 10% of the results obtained from SP docking. The QED was utilized to assess the drug-likeness of small molecules, retaining those with a QED score greater than 0.5. Potential pan-assay interference compounds (PAINS) were eliminated to minimize the likelihood of false positive results [83]. The MMGBSA was also used for the calculation of binding free energy between ligands and receptor molecules. PyMol software (version 2.5.5) was utilized for the docking analysis and visualization.

### 4.7. Cell Experiments

Cell viability was determined by CCK-8 assay (Yeasen, Shanghai, China), and was performed as previously described [84].

## Figures and Tables

**Table 1:** Multivariate Cox analysis.

ID	Coef	HR	HR.95L	HR.95H	*p*-Value
ACSS2	−0.3552	0.7010	0.4379	1.1222	0.1389
C2CD2	0.7737	2.1677	1.3553	3.4672	0.0012
CXCL9	−0.2091	0.8113	0.7176	0.9173	0.0008
KRT15	−0.1919	0.8254	0.7122	0.9566	0.0108
MRPL13	0.3990	1.4903	1.0068	2.2061	0.0462
NR3C2	−0.4672	0.6268	0.3745	1.0491	0.0755
PGK1	0.6295	1.8767	1.2022	2.9297	0.0056
PIGR	−0.2308	0.7939	0.6619	0.9522	0.0129
RBP4	0.2766	1.3187	1.0548	1.6486	0.0152
SORBS1	−0.4613	0.6304	0.4339	0.9161	0.0155

HR, hazard ratio; Coef, coefficient.

**Table 2:** Four compounds matched the selected condition.

	CAS No.	Structure	Name	Formula	Docking Score	dGBind (kcal/mol)
D715-2871	/	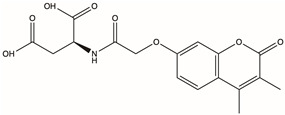	2-{2-[(3,4-dimethyl-2-oxo-2H-chromen-7-yl)oxy]acetamido}butanedioic acid	C_17_H_17_NO_8_	−16.709	−53.47
Y040-8304	957001-29-5	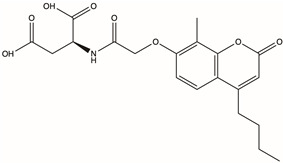	2-{2-[(4-butyl-8-methyl-2-oxo-2H-chromen-7-yl)oxy]acetamido}butanedioic acid	C_20_H_23_NO_8_	−16.417	−89.62
D715-0344	/	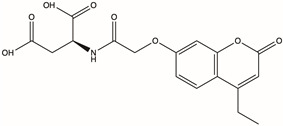	2-{2-[(4-ethyl-2-oxo-2H-chromen-7-yl)oxy]acetamido}butanedioic acid	C_17_H_17_NO_8_	−16.314	−87.54
D231-0058	775298-43-6	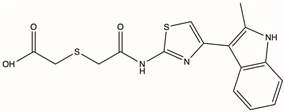	2-[({[4-(2-methyl-1H-indol-3-yl)-1,3-thiazol-2-yl]carbamoyl}methyl)sulfanyl]acetic acid	C_16_H_15_N_3_O_3_S_2_	−16.232	−77.19

## Data Availability

The original data presented in the study are openly available in TCGA at https://www.cancer.gov/ccg/research/genome-sequencing/tcga (accessed on 1 April 2022) and GEO (GSE32641, GSE36295, GSE42568, GSE53752, and GSE139038).

## Supplementary Materials

The following supporting information can be downloaded at: https://www.mdpi.com/article/10.3390/ph17121636/s1, Figure S1: RNA levels of ten risk genes in GEO datasets; Figure S2: (A–C) The risk curve of every sample arranged by risk score in the training (A), test (B), and entire (C) sets. (D–F) The scatter plot of survival overview of patients with breast cancer in the training (D), test (E), and entire (F) sets. The red dot represents death, and the green dot represents survival; Figure S3: The alignments of ADP (carbon skeletal structure colored with yellow) with D715-2871 (A), Y040-8304 (B), D715-0344 (C), and D231-0058 (D) in the pocket of PGK1 (2X13). Table S1. The information of HER2+, Luminal A, Luminal B and triple-negative subtype tumor samples in the GSE datasets. Table S2. The information of GSE datasets used in this study. Table S3. Results of GO enrichment analysis. Table S4. Results of KEGG enrichment analysis.
