# Supplementary material for: Identification of a Potential PGK1 Inhibitor with the Suppression of Breast Cancer Cells Using Virtual Screening and Molecular Docking
Source: Pharmaceuticals (Basel). 2024 Dec 5;17(12):1636. doi: 10.3390/ph17121636 (PMC11676932; doi:10.3390/ph17121636)
Supplement: Supplementary file 1 [file pharmaceuticals-17-01636-s001.zip › pharmaceuticals-3339364-supplementary.pdf]

pharmaceuticals

MDPI

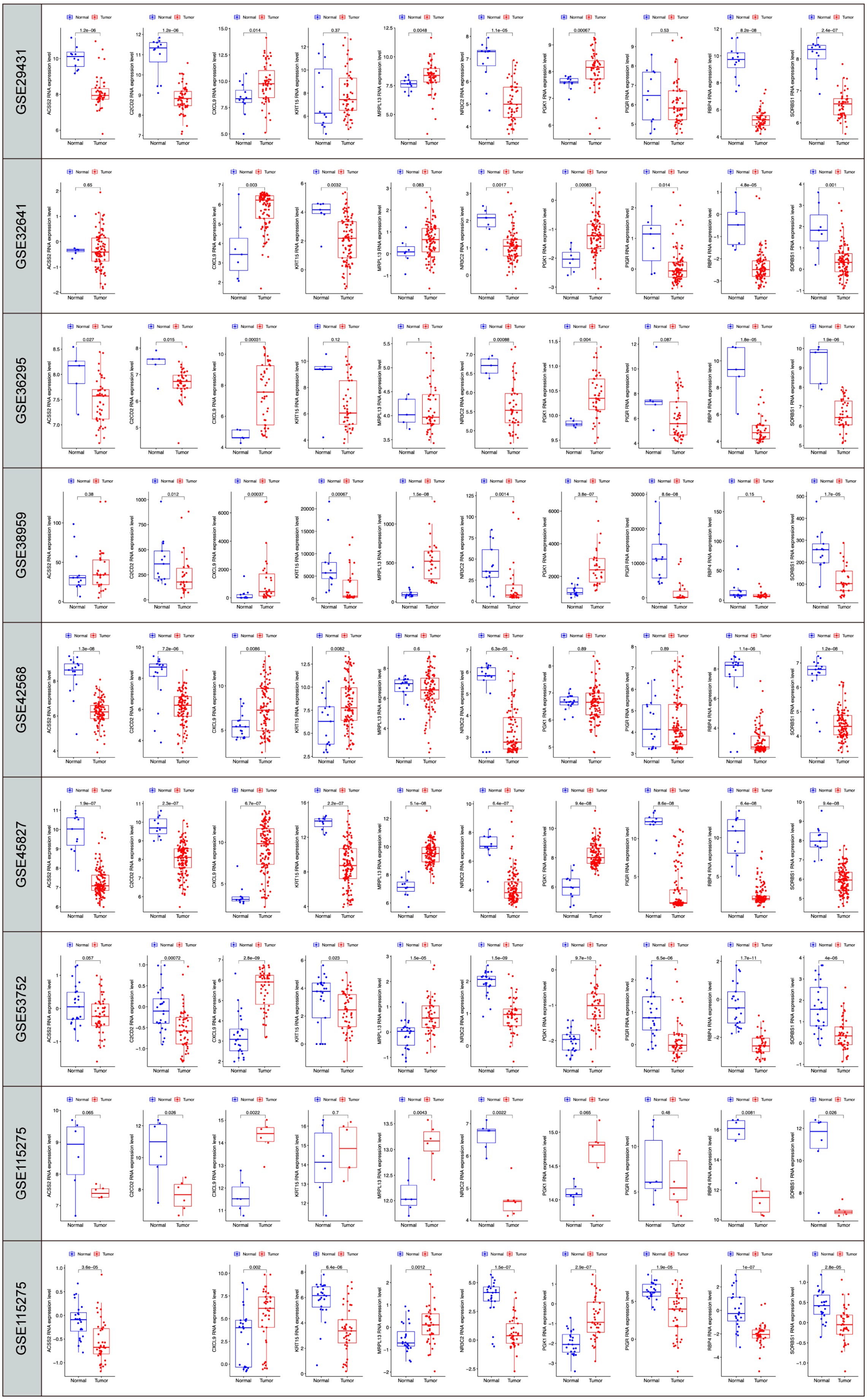

Figure S1. RNA levels of ten risk genes in GEO datasets.

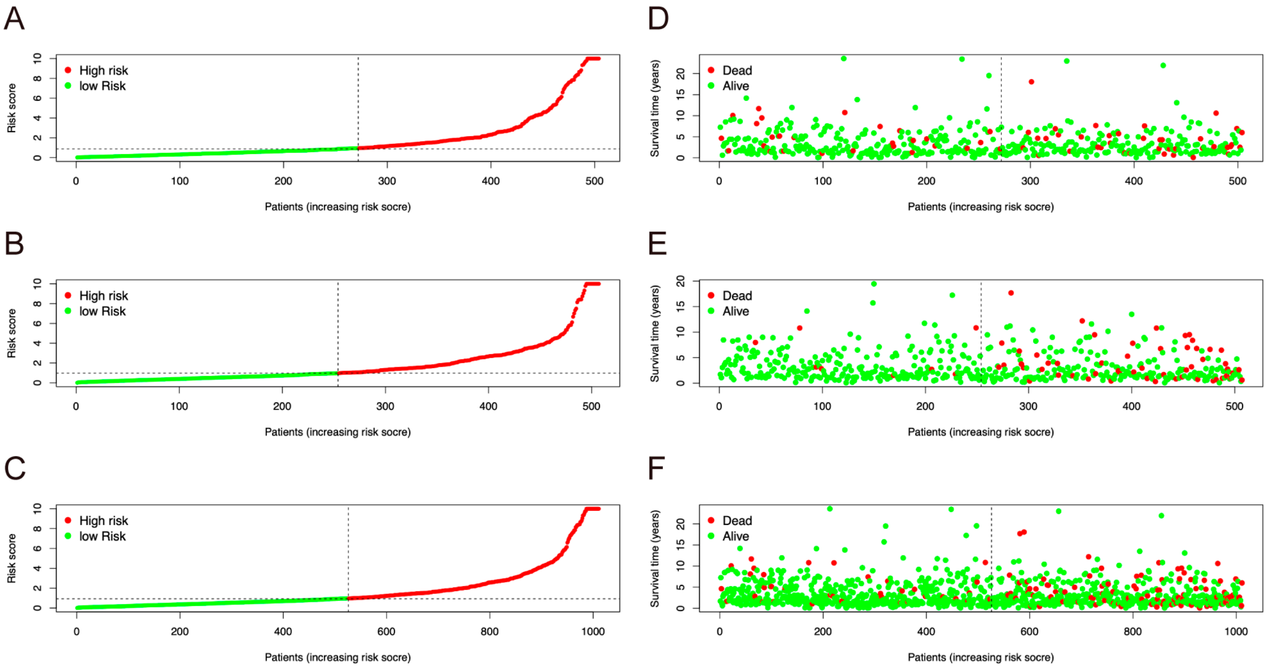

Figure S2. (A-C) The risk curve of every sample arranged by risk score in the training (A), test (B) and entire (C) sets. (D-F) The scatter plot of survival overview of patient with breast cancer in the training (D), test (E) and entire (F) sets. The red dot represents death, and the green dot represents survival.

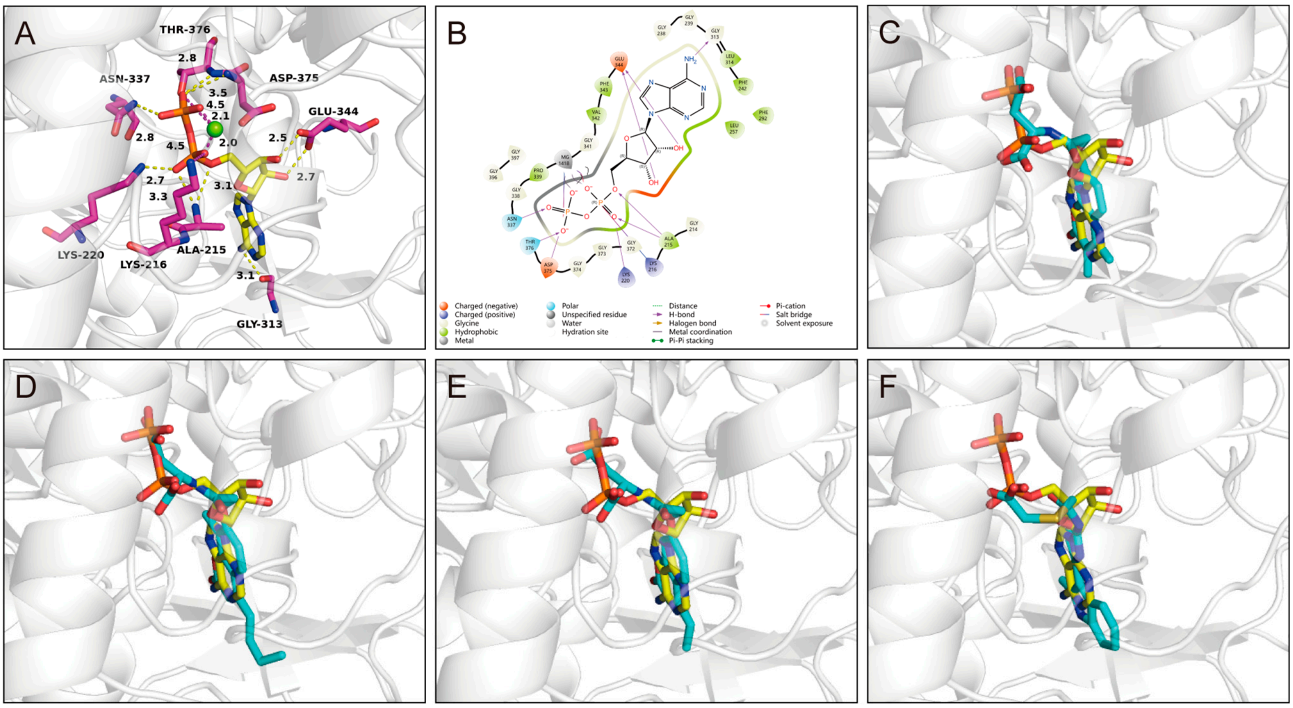

Figure S3. The alignments of ADP (carbon skeletal structure colored with yellow) with the potential PGK1 inhibitor. (A,B) The interaction between PGK1 (2X13) and ADP. (B) D715-2871. (C) Y040-8304, (D) D715-0344, and (E) D231-0058.

Table S1. The information of HER2+, Luminal A, Luminal B and triple-negative subtype tumor samples in the GSE datasets.

Subtypes	GSE number	Normal	Tumor
HER2+	GSE29431	12	48
	GSE45827	11	30
	GSE65194	11	39
Luminal A	GSE45827	11	29
	GSE65194	11	29
Luminal B	GSE45827	11	30
	GSE65194	11	30
triple-negative	GSE38959	13	30
	GSE45827	11	41
	GSE65194	11	55
	GSE115275	6	6

Table S2. The information of GSE datasets used in this study.

GSE accession	Platform	Samples	
		Normal	Tumor
**GSE29431**	GPL570	12	54
GSE38959	GPL4133	12	30
GSE45827	GPL570	11	130
GSE65194	GPL570	11	153
GSE115275	GPL21827	6	6
Total		51	357
