# Supplementary material for: Identification of a Potential PGK1 Inhibitor with the Suppression of Breast Cancer Cells Using Virtual Screening and Molecular Docking
Source: Pharmaceuticals (Basel). 2024 Dec 5;17(12):1636. doi: 10.3390/ph17121636 (PMC11676932; doi:10.3390/ph17121636)
Supplement: Supplementary file 1 [file pharmaceuticals-17-01636-s001.zip › Table S1.pdf]

Table S1. The information of HER2+, Luminal A, Luminal B and triple-negative subtype tumor samples in the GSE datasets.

Subtypes	GSE number	Normal	Tumor
HER2+	GSE29431	12	48
	GSE45827	11	30
	GSE65194	11	39
Luminal A	GSE45827	11	29
	GSE65194	11	29
Luminal B	GSE45827	11	30
	GSE65194	11	30
triple-negative	GSE38959	13	30
	GSE45827	11	41
	GSE65194	11	55
	GSE115275	6	6
