# Supplementary material for: Identification of a Potential PGK1 Inhibitor with the Suppression of Breast Cancer Cells Using Virtual Screening and Molecular Docking
Source: Pharmaceuticals (Basel). 2024 Dec 5;17(12):1636. doi: 10.3390/ph17121636 (PMC11676932; doi:10.3390/ph17121636)
Supplement: Supplementary file 1 [file pharmaceuticals-17-01636-s001.zip › Table S2.pdf]

Table S2. The information of GSE datasets used in this study.

GSE accession	Platform	Samples	
		Normal	Tumor
**GSE29431**	GPL570	12	54
GSE38959	GPL4133	12	30
GSE45827	GPL570	11	130
GSE65194	GPL570	11	153
GSE115275	GPL21827	6	6
Total		51	357
