# Supplementary figures and images for: Identification of a Potential PGK1 Inhibitor with the Suppression of Breast Cancer Cells Using Virtual Screening and Molecular Docking
Source: Pharmaceuticals (Basel). 2024 Dec 5;17(12):1636. doi: 10.3390/ph17121636 (PMC11676932; doi:10.3390/ph17121636)

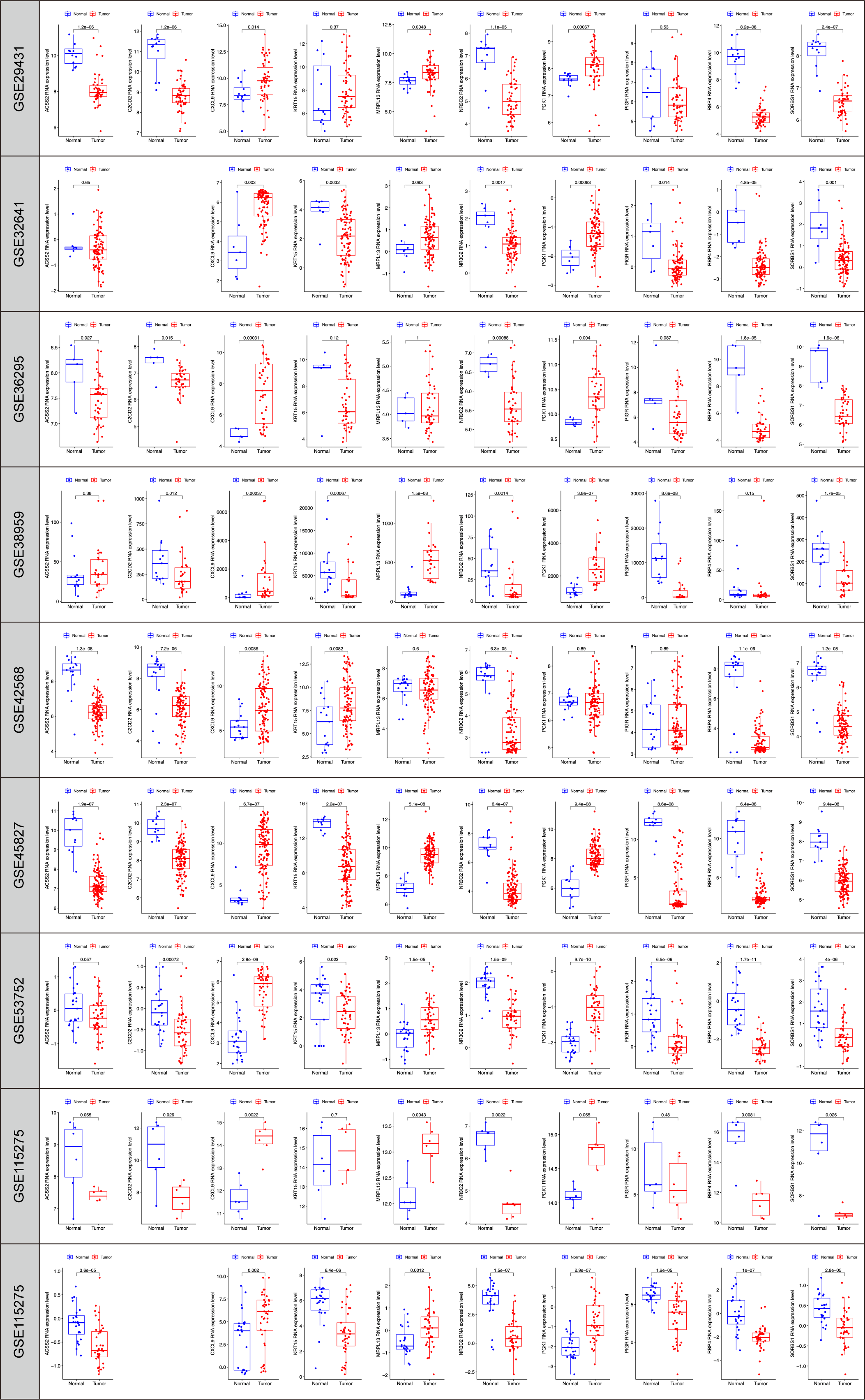

Supplement: Supplementary file 1 [file pharmaceuticals-17-01636-s001.zip › Figure S1.tif]

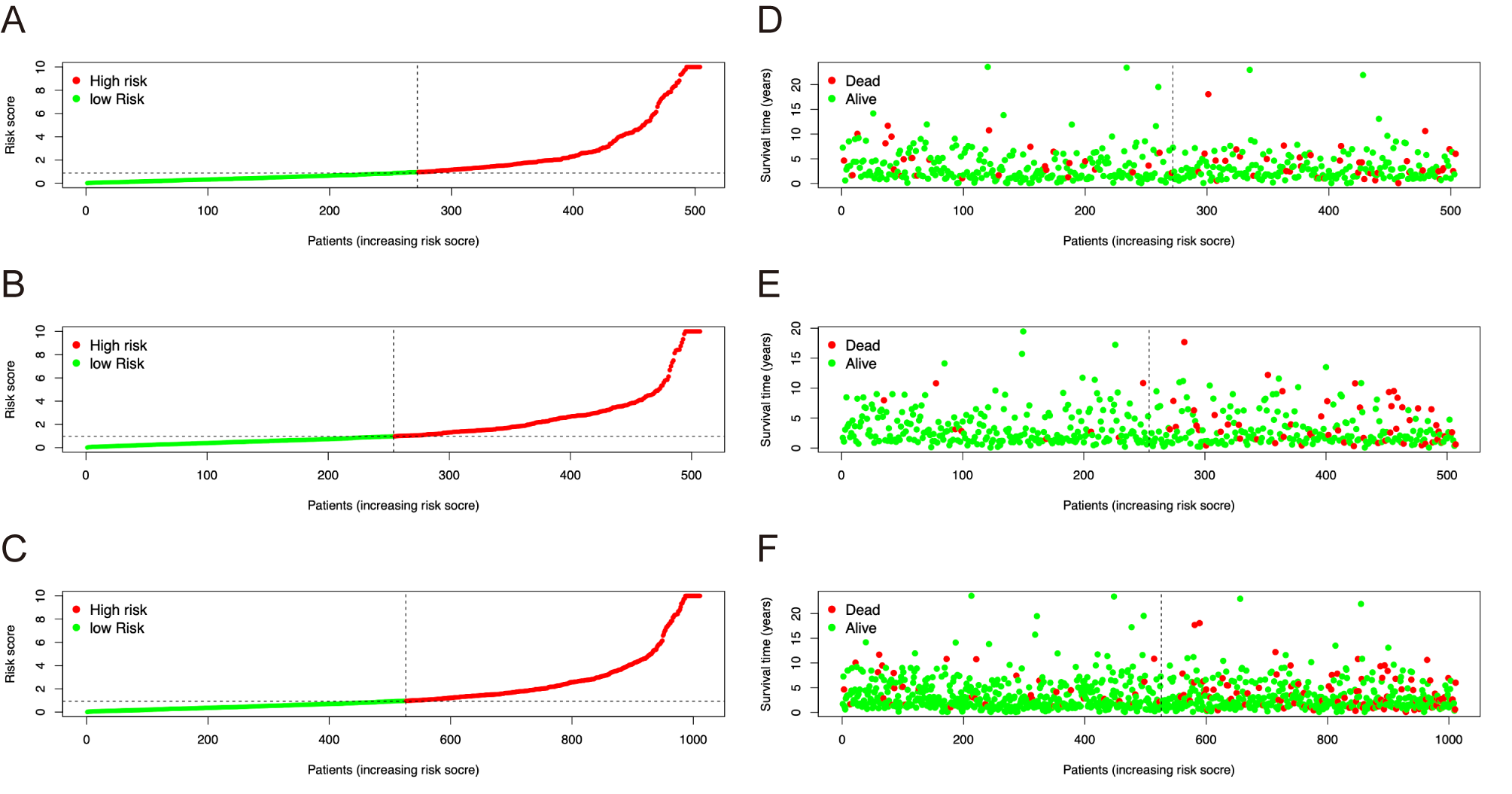

Supplement: Supplementary file 1 [file pharmaceuticals-17-01636-s001.zip › Figure S2.tif]

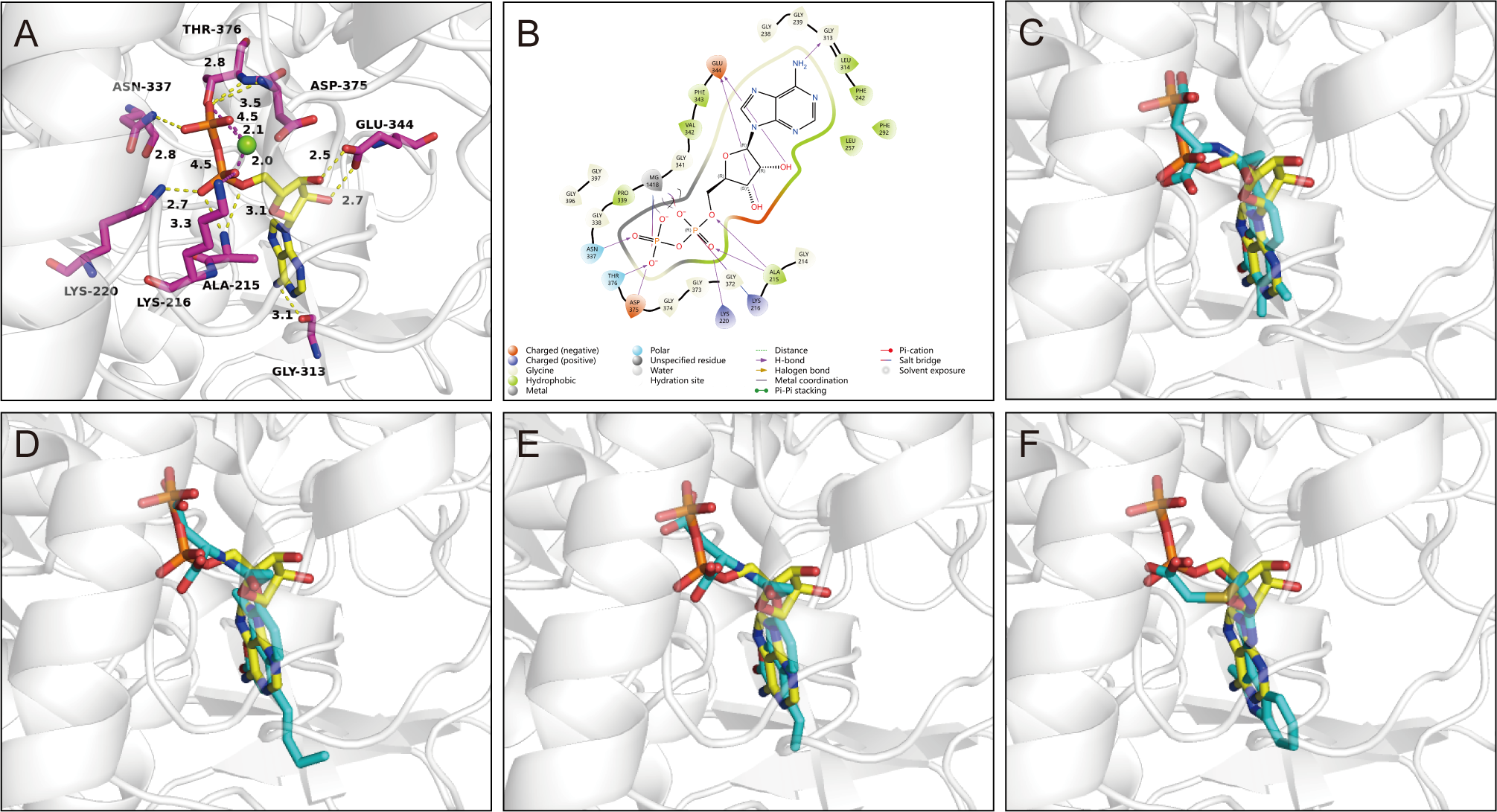

Supplement: Supplementary file 1 [file pharmaceuticals-17-01636-s001.zip › Figure S3.tif]
